# Effects of Bisphenol A and Its Alternatives, Bisphenol F and Tetramethyl Bisphenol F on Osteoclast Differentiation

**DOI:** 10.3390/molecules26206100

**Published:** 2021-10-09

**Authors:** Hye-Min Kim, Seon-Min Lee, Jungil Choi, Nak-Kyun Soung, Jeong-Doo Heo

**Affiliations:** 1 Biological Resources Research Group, Bioenvironmental Science and Toxicology Division, Gyeongnam Branch Institute, Korea Institute of Toxicology (KIT), 17 Jegok-gil, Munsan-eup, Jinju-si 52834, Gyeongsangnam-do, Korea; hyemin.kim@kitox.re.kr (H.-M.K.); smlee84@kitox.re.kr (S.-M.L.); jungil.choi@kitox.re.kr (J.C.); 2 Anticancer Agent Research Center, Korea Research Institute of Bioscience and Biotechnology, 30 Yeongudanji-ro, Ochang-eup, Cheongwon-gu, Cheongju-si 28116, Chungcheongbuk-do, Korea; soungnak@kribb.re.kr

**Keywords:** bisphenol A, bisphenol F, tetramethyl bisphenol F, osteoclast differentiation, bone remodeling, xenoestrogen

## Abstract

Bisphenol A (BPA) is a typical environmental endocrine disruptor that exhibits estrogen-mimicking, hormone-like properties and can cause the collapse of bone homeostasis by an imbalance between osteoblasts and osteoclasts. Various BPA substitutes, structurally similar to BPA, have been used to manufacture ‘BPA-free’ products; however, the regulatory role of BPA alternatives in osteoclast differentiation still remains unelucidated. This study aimed to investigate the effects of these chemicals on osteoclast differentiation using the mouse osteoclast precursor cell line RAW 264.7. Results confirmed that both BPA and its alternatives, bisphenol F and tetramethyl bisphenol F (TMBPF), were nontoxic to RAW 264.7 cells. In particular, tartrate-resistant acid phosphatase (TRAP)-positive multinucleated cell staining and activity calculation assays revealed that TMBPF enhanced osteoclast differentiation upon stimulation of the receptor activator of nuclear factor-kappa B ligand (RANKL). Additionally, TMBPF activated the mRNA expression of osteoclast-related target genes, such as the nuclear factor of activated T-cells, cytoplasmic 1 (*NFATc1*), tartrate-resistant acid phosphatase (*TRAP*), and cathepsin K (*CtsK*). Western blotting analysis indicated activation of the mitogen-activated protein kinase signaling pathway, including phosphorylation of c-Jun N-terminal kinase and p38. Together, the results suggest that TMBPF enhances osteoclast differentiation, and it is critical for bone homeostasis and skeletal health.

## 1. Introduction

Bone homeostasis is regulated by the balance between bone forming by osteoblasts and bone resorbing by osteoclasts [1]. An imbalance in bone homeostasis causes bone-related diseases, such as osteoporosis, periprosthetic osteolysis, rheumatoid arthritis, and Paget’s disease [2,3,4,5]. Osteoclasts are large, multinucleated cells in charge of the dissolution and absorption of bone derived from the monocyte–macrophage lineage of hematopoietic stem cells [6]. The binding of the receptor activator of nuclear factor-kappa B ligand (RANKL) to its receptor RANK on osteoclast precursor cell membranes stimulates the activation of downstream signaling pathways, including the nuclear factor-kappa B (NF-κB) pathway and mitogen-activated protein kinases (MAPKs), such as extracellular signal-regulated kinase (ERK), c-Jun N-terminal kinase (JNK), and p38 [7,8,9,10,11]. Activation of these signaling pathways modulates autoamplification of the nuclear factor of activated T-cells, cytoplasmic 1 (NFATc1), and the maturation effect of osteoclast target genes, such as tartrate-resistant acid phosphatase (TRAP) and cathepsin K (CtsK) [12,13,14].

Estrogen is an important regulator of bone metabolism in both men and women [15]. Estrogen deficiency is associated with a gap between bone resorption and bone formation and causes bone loss, which, in turn, can be prevented by estrogen replacement therapy [16,17,18]. The role of estrogen in bone homeostasis is clearly illustrated by the inhibition of bone resorption by estrogen via direct induction of apoptosis in osteoclasts [19]. Estrogen acts through two receptors, namely estrogen receptor alpha (ERα) and estrogen receptor beta (ERβ). Conditional ablation of ERα in osteoclasts protects against ovariectomy (OVX)-induced bone loss [20].

Bisphenol A (BPA) is one of the most produced chemicals worldwide. It is widely used in the production of polymer plastics and coatings, mainly polycarbonate and epoxy resins [21]. However, it is one of the major contributors to endocrine disruption in humans and animals [22]. The biological effect of BPA is exerted by xenoestrogens, which exhibit estrogen-mimicking hormone-like properties [23,24]. BPA has been found to bind to the two nuclear estrogen receptors (ERs), ERα and ERβ [25,26]. Moreover, it blocks natural hormones, causing human health problems, including alterations in sperm fertility, abnormalities in sex organs, altered immune function, cancers, diabetes, obesity, and cardiovascular diseases [27,28,29,30,31,32]. For this reason, BPA has been banned for use in several countries.

Alternatively, several BPA substitutes, such as bisphenol S (BPS), bisphenol F (BPF), and tetramethyl bisphenol F (TMBPF), are currently being used in various products, labeled as ‘BPA-free’ products [33,34]. This term gives the impression that these products are safe and that BPA substitutes are widely used; however, the safety aspect of these substitutes has not been fully verified [35,36]. Given the xenoestrogen and antiandrogenic activities of BPA, several studies have demonstrated similar effects of BPA substitutes as well [37,38]. BPA and BPF showed estrogenic activities in a yeast two-hybrid system, and BPF has exhibited an estrogen agonistic effect in vivo [39,40].

This study aimed to investigate the effects of BPA and its substitutes, BPF and TMBPF, on osteoclast differentiation. Mouse leukemic monocyte/macrophage cell line RAW 264.7 was used to analyze the role of BPA alternatives in osteoclasts and reveal the underlying mechanisms. Our results unraveled the potential of a novel endocrine-disrupting chemical TMBPF in enhancing osteoclast differentiation and causing bone-related diseases.

## 2. Results and Discussion

### 2.1. Effects of Bisphenol A and Its Alternatives on Cell Viability in Mouse Bone Marrow Macrophage RAW 264.7 Cells

**Figure 1:**
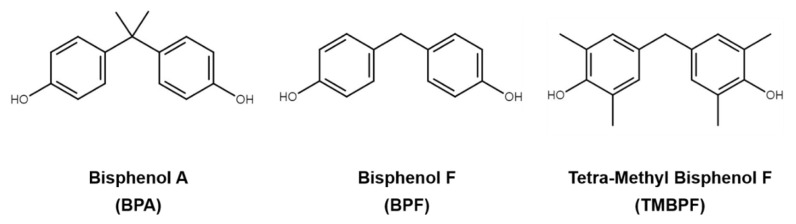
Chemical structures of bisphenol A (BPA) and its substitutes, bisphenol F (BPF) and tetramethyl bisphenol F (TMBPF).

Maintaining bone homeostasis by balancing osteoclasts and osteoblasts is important for bone health, and estrogen plays an important role in the regulation of bone turnover by targeting RANKL expression [41]. BPA is a representative, endocrine-disrupting chemical that mimics estrogen to alter the systemic hormonal regulation of the bone homeostasis and bone remodeling process. Several previous studies have reported the skeletal effects of BPA both in vitro and in vivo [42]. Treatment with BPA reduced osteoblast and bone formations by MC3T3-E1 preosteoblasts, and low-dose exposure to BPA reduced femur stiffness in female offspring [43,44]. Several BPA alternatives that are structurally similar to BPA, such as BPF and TMBPF, are widely used in “BPA-free”-labeled products (Figure 1). However, the effects of BPF and TMBPF on bone remodeling are still relatively unknown.

**Figure 2:**
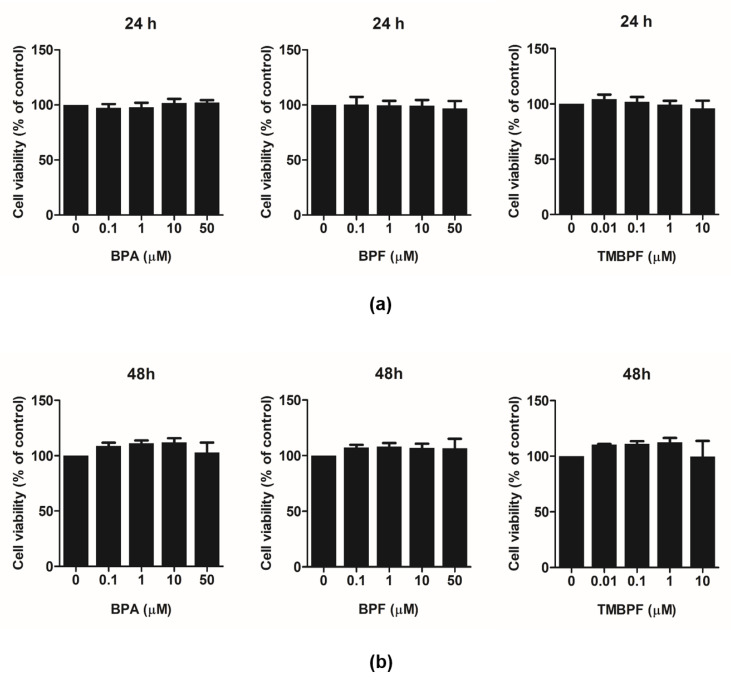
Effect of BPA, BPF, and TMBPF on the viability in RAW 264.7 cells. Cells were treated and incubated with compounds for (**a**) 24 h and (**b**) 48 h, and cell viability was measured via cell counting kit-8 (CCK-8) kit.

In this study, we first examined the cell viability of mouse osteoclast precursor cells, RAW 264.7, in order to test the effect of BPA and its substitutes on osteoclast differentiation. Cells were incubated in the presence of BPA, BPF, and TMBPF at the indicated concentrations for 24 and 48 h. As a result, their proliferation was not diminished even after treatment with 50 μM BPA and BPF. Cells treated with TMBPF also showed no reduction in cell viability (Figure 2). Therefore, the data suggested that none of the three bisphenol chemicals caused any serious cytotoxicity in RAW 264.7 cells.

A previous study had reported that BPA significantly inhibits RANKL-induced osteoclast differentiation in RAW 264.7 cells [44]. It confirmed that cell viability decreased by 20% with 12.5 μM BPA and that BPA causes apoptosis by suppression of B-cell lymphoma 2 and activation of caspase 3 and caspase 8 in RAW 264.7 cells. However, our data showed no BPA cytotoxicity, even at a concentration of 50 μM, which was inconsistent with previous reports.

### 2.2. Activation of Osteoclast Differentiation by Bisphenol A and Its Substitutes

**Figure 3:**
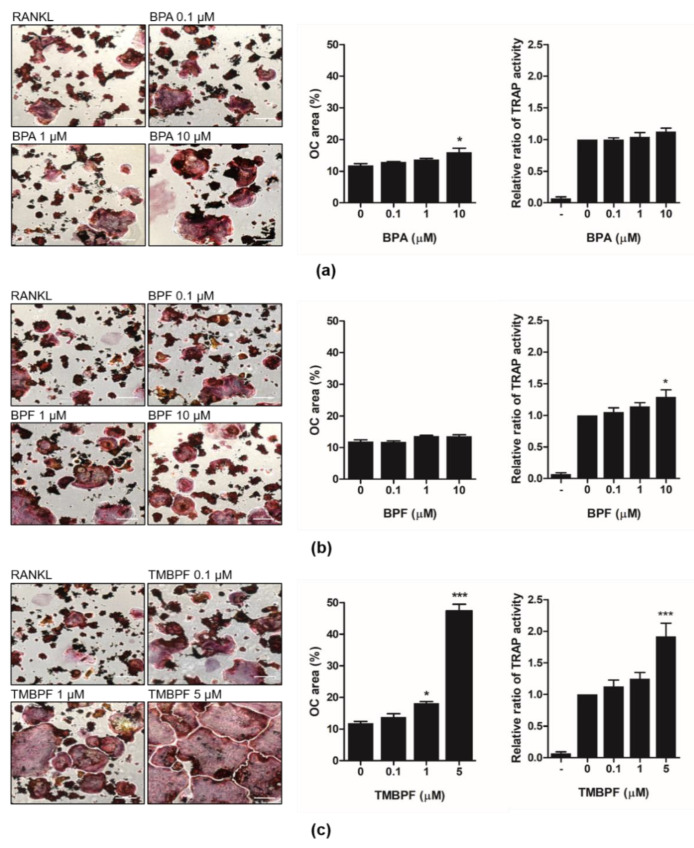
Effect of BPA, BPF, and TMBPF on osteoclast differentiation in mouse osteoclast precursor RAW 264.7 cells. RAW 264.7 cells were cultured with a complete medium containing RANKL (50 ng/mL) in the presence of different concentrations of (**a**) BPA, (**b**) BPF, and (**c**) TMBPF for 3 days. Cells were stained using a tartrate-resistant acid phosphatase (TRAP) staining kit. The area (%) of TRAP-positive multinucleated osteoclasts was quantified. The relative ratio of TRAP activity was measured at 405 nm. The (-) group refers to no RANKL treatment. All experiments were performed in triplicates. * *p* < 0.5, *** *p* < 0.001 vs. control. Scale bar: 200 µm.

TRAP is an important histochemical osteoclast marker and is recognized to be a molecule with functions in bone resorption [45]. To elucidate the effect of BPA and its alternatives on osteoclast differentiation, RANKL-induced osteoclast differentiation, TRAP staining, and a TRAP activity assay were performed. RAW 264.7 cells were incubated with RANKL (50 ng/mL) with bisphenol A and its substitutes (BPF and TMBPF) for 3 days. TRAP-positive staining results indicated high concentrations of TMBPF (5 μM) to dramatically activate RANKL-induced osteoclast differentiation than RANKL control. TRAP-positive multinuclei osteoclast area (%) and TRAP activity also dramatically increased after treatment with 5 μM of TMBPF (Figure 3c).

The skeletal effects of BPA and its substitutes are heterogenous [42]. BPA inhibits osteoblast formation and induces apoptosis of osteoblasts and osteoclasts in vitro [44]. Despite in vivo actions, BPA decreases bone strength and bone mineral content in female rodents but increases it in male rodents prenatally [46,47]. Bisphenol AF encourages osteoblast formation, but BPS inhibits osteoblast formation [48].

Our data revealed that treatment with BPA slightly increased osteoclast differentiation, although this was not significant using the TRAP activity assay (Figure 3a). Treatment with BPF also slightly increased TRAP activity, although there was no difference in TRAP-positive staining (Figure 3b). However, a high concentration (50 μM) of BPA or BPF was not cytotoxic, and the hypothesis that treatment with high concentrations of these compounds can enhance osteoclast differentiation may be considered.

### 2.3. Effect of TMBPF on Osteoclast Target-Related Genes Expression

**Figure 4:**
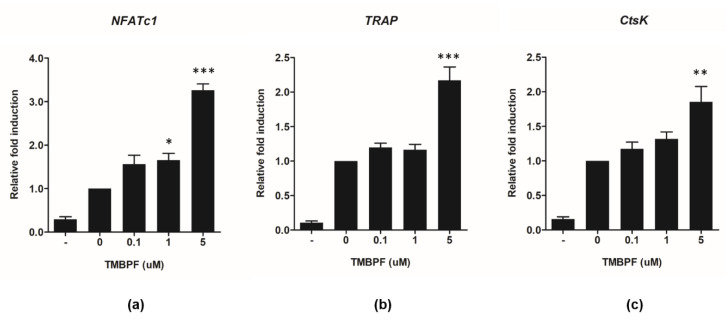
TMBPF activates osteoclast-specific gene expression. RAW 264.7 cells were incubated with RANKL (50 ng/mL) with various concentrations of TMBPF (0.1, 1, 5 μM). (-) group refers to no RANKL treatment. The expression levels of osteoclasts-related marker genes, including (**a**) *NFATc1*, (**b**) *TRAP*, and (**c**) *CtsK*, were detected by qRT-PCR. Results were normalized to the expression of β-actin. * *p* < 0.5, ** *p* < 0.01, *** *p* < 0.001 vs. 0 group.

Next, we examined the effects of TMBPF on mRNA expression of osteoclast differentiation-related marker genes, such as *NFATc1*, *TRAP*, and *CtsK*, using quantitative reverse transcription-polymerase chain reaction PCR (qRT-PCR). RAW 264.7 cells were incubated in alpha-MEM medium in the presence of TMBPF for 3 days, and total RNA was extracted subsequently for analysis. The mRNA levels of *NFATc1*, which encodes a key transcription factor involved in osteoclast differentiation, were activated after treatment with 5 μM TMBPF (Figure 4a), and those of other osteoclast target genes, *TRAP* and *CtsK*, also increased upon treatment with TMBPF (Figure 4b,c). The results together suggested that TMBPF stimulates osteoclast differentiation through NFATc1 and the activation of downstream osteoclast target genes.

### 2.4. Acceleration of the JNK and p38 Pathways by TMBPF in RAW 264.7 Cells

**Figure 5:**
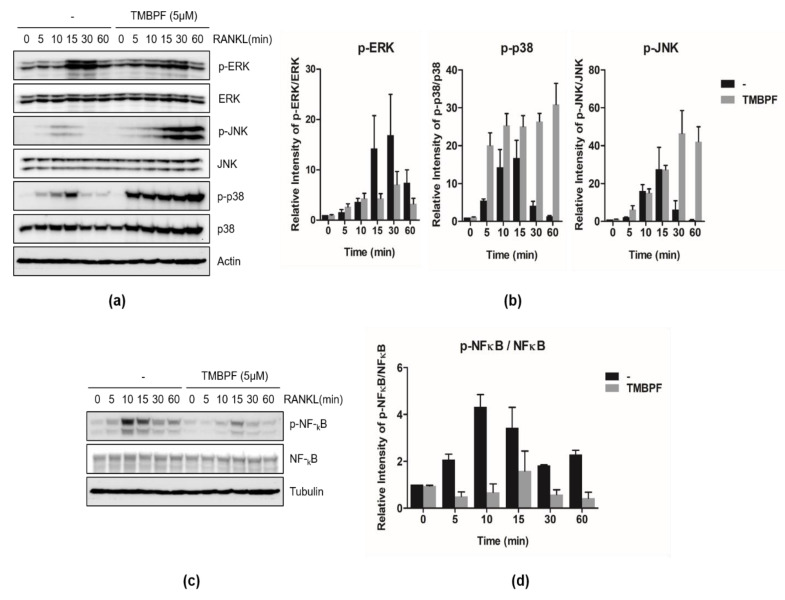
Effect of TMBPF on MAKP kinase and the NF-kB signaling pathway. RAW 264.7 cells were incubated with RANKL (50 ng/mL) and in presence or absence of TMBPF (5 μM) for indicated times (0, 5, 10, 15, 30, 60 min). (**a**,**c**) Phosphorylation of ERK, JNK, p38, and NF-kB was determined by western blotting. The measurement of intensity p-ERK, p-JNK, p-p38, and p-NF-kB were normalized by (**b**) β-actin and (**d**) α-tubulin.

Binding of RANK to RANKL induces the activation of signaling cascades for osteoclast differentiation; TNF receptor-associated factor 6 (TRAF6) transmits the RANK/RANKL signal to downstream targets, such as ERK, JNK, p38, NF-κB, and NFATc1 [49]. To determine which signaling pathways are involved in TMBPF-triggered osteoclast differentiation, we investigated the effect of TMBPF on intracellular signaling pathways in RANKL-induced RAW 264.7 cells. Cells were treated with RANKL, with or without TMBPF, for the indicated durations. Results showed that phosphorylation of JNK and p38 was maintained continuously up to 60 min with 5 μM TMBPF treatment compared to that in RANKL control; however, no increases in ERK phosphorylation were observed, even after TMBPF treatment (Figure 5a,b).

We further investigated the effect of TMBPF on the NF-κB pathway, which is another major signaling pathway involved in RANKL-induced osteoclast differentiation. TMBPF-treated RAW 264.7 cells exhibited significantly decreased activation of NF-κB phosphorylation compared to RANKL control after 10 to 15 min of RANKL treatment (Figure 5c,d). The data suggested that activation of osteoclast differentiation upon TMBPF treatment is mediated by JNK and p38 signaling pathways, but not by ERK and NF-κB activation.

TMBPF is one of the currently used BPA alternatives and it is used in the new epoxy-coated products, such as metal beverage and food cans [50]. TMBPF was selected using a ‘safety by design’ approach, to exhibit the same properties as BPA, without the estrogenic effects. It was shown to not produce any estrogenic effects in both in vitro and in vivo [33,51]. According to a recent study, TMBPF has effects on adipogenesis and fat accumulation, having nonestrogenic, antiadipogenic effects [52]. Moreover, TMBPF revealed antiestrogenic and antiandrogenic activity in several cancer cell lines [33,53]. However, very limited independent research has been performed on TMBPF, and its effects on bone homeostasis had not been investigated. In this study, we presented the significant activation effects of TMBPF on osteoclast differentiation in RAW 264.7 cells (Figure 3). This activation is through the MAPK signaling pathways, including JNK and p38 (Figure 5). It suggests the possibility of the xenoestrogen effect of TMBPF, such as BPA; however, further investigation of the mechanism of estrogenic and skeletal effects of TMBPF would be required in the future.

## 3. Materials and Methods

### 3.1. Reagents

BPA and BPF were obtained from Sigma-Aldrich (St. Louis, MO, USA), and TMBPF was purchased by Toronto Research Chemicals (Toronto, ON, Canada). Culture media, fetal bovine serum (FBS), and antibiotics were from Gibco (Carlsbad, CA, USA). The cell counting kit-8 (CCK-8) was purchased from Dojindo (Rockville, MD, USA). Recombinant mouse RANKL was from PeproTech (JN, USA). All primary antibodies were purchased from Cell Signaling Technology (Danvers, MA, USA).

### 3.2. Cell Culture and Osteoclast Differentiation

The murine macrophages, RAW 264.7 cells, which have the potential to differentiate into osteoclasts, were obtained from the American Type Culture Collection (ATCC, Manassas, VA, USA). The cells were maintained in Dulbecco’s Modified Eagle’s Medium (DMEM) supplemented with 10% FBS and 1% penicillin/streptomycin (Gibco, USA) in a humidified incubator at 37 °C supplied with 5% CO_2_. To differentiate to osteoclasts, RAW 264.7 cells were seeded in 96-well plates at 2 × 10^3^ cells/well. After 24 h, the cells were incubated with recombinant mouse RANKL in the absence or presence of the indicated concentrations of BPA, BPF, and TMBPF for 3 days in α-MEM containing 10% FBS with a change of medium every 2 days.

### 3.3. Cell Viability Assays

RAW 264.7 cells were seeded in 96-well plates at a density of 5 × 10^3^ cells/well and cultured with various concentrations of BPA, BPF (0.1, 1, and 10 µM), and TMBPF (0.1, 1, and 5 µM) for 24 and 48 h. After that, the culture medium was replaced with fresh medium containing CCK-8 and incubated for 2 h at 37 °C with 5% CO_2_. The plates were analyzed at 450 nm using the Synergy HTX multimode reader (BioTeK Instruments, VT, USA).

### 3.4. TRAP Staining and Activity Assays

Multinucleated osteoclasts were washed with PBS, fixed with 4% paraformaldehyde for 15 min, and then stained with the Acid Phosphatase, Leukocyte (TRAP) kit (Sigma-Aldrich, MO, USA) according to the manufacturer’s instructions. TRAP-positive multinucleated cells were visualized under a microscope, and the percentage of osteoclast area was calculated. To measure TRAP activity, cells were washed with physiological saline and lysed with extraction buffer (physiological saline including 1% NP-40), and then incubated with reaction buffer (0.5 M sodium tartrate buffer, pH 5.2, 0.5 M sodium acetate, 12.5 mM pNPP). After 1 h, an equal volume of stop solution buffer (0.5 N NaOH) was added to the reaction. Absorbance was measured at 405 nm after color formation. TRAP activity was presented as the relative ratio of the control.

### 3.5. RNA Preparation and RNA Quantitation by RT-PCR

Total RNA was isolated using the RNeasy Mini Kit (Qiagen, Germany) according to the manufacturer’s instructions. Purity and concentration of the RNA were analyzed by NanoDrop (DeNovix, DE, USA). Total RNA (2 µg) was reverse transcribed into cDNA using the QuantiNova reverse transcription kit (Qiagen, Germany). Real-time quantitative polymerase chain reaction (qRT-PCR) was conducted using the Thermal Cycler Dice Real-Time System III (TP950) (TAKARA, Japan) with the GoTaq^®^ qPCR Master Mix (Promega, WI, USA) under the following conditions: 40 cycles of denaturation 15 s at 95 °C and amplification 1 min at 60 °C. Data were analyzed by the 2^−ΔΔCT^ method, and gene expression was normalized to β-actin. Primer sets were: mouse NFATc1 (5′-CCGTTGCTTCCAGAAAATAACA-3′ and 5′-TGTGGGATGTGAACTCGGAA-3′), mouse TRAP (5′-ACTTCCCCAGCCCTTACTAC-3′ and 5′-ACATAGCCCACACCGTTCTC-3′), mouse cathepsin K (5′-CTTCCAATACGTGCAGCAGA-3′ and 5′-GTGCTTGCTTCCCTTCTGG-3′), and mouse β-actin (5′-AGCCATGTACGTAGCCATCC-3′ and 5′-CTCTCAGCTGTGGTGGTGA-3′.

### 3.6. Immunoblot Analysis

RAW 264.7 cells were treated with mouse recombinant RANKL (50 ng/mL) for indicated times of TMBPF (5 µM) GoTaq^®^ qPCR Master Mix. Cells were lysed in lysis buffer (PBS plus 0.5% NP-40, protease inhibitor) and centrifuged at 13,500 rpm for 20 min, and the supernatants were subsequently collected. Equal amounts of supernatant with 4× sample buffer (LPS solution, Korea) were separated by 8% to 10% SDS-PAGE and then transferred to PVDF membranes. The membranes were blocked in 5% skim milk/TBST and incubated with the indicated antibodies. The membrane was washed and incubated with HRP-conjugated secondary antibody in 5% skim milk/TBST, followed by detection using the ChemiDoc Imaging System (Bio-Rad, CA, USA). Primary antibodies used were: p-ERK (#4370), ERK (#4695), p-JNK (#9255), JNK (#9252), p-p38 (#4511), p38 (#8690), p-NF-κB (#3031), NF-κB (#8242), α-tubulin (#2144), and β-actin (#3700). All antibodies are purchased from Cell Signaling Technology (Danvers, MA, USA).

### 3.7. Statistical Analysis

Data are presented as the mean and standard deviation of three experiments. Statistical significance was analyzed using one-way or two-way analysis of variance (ANOVA) for comparisons between the two means of two or more groups. A value of *p* < 0.05 was considered statistically significant. GraphPad Prism software was used for data analysis.

## 4. Conclusion

Bisphenol A is a major endocrine disruptor that plays the role of a xenoestrogen through mimicking estrogen by interacting with two estrogen receptors. It causes an imbalance between osteoclasts and osteoblasts, leading to bone-related diseases. However, BPA alternatives on xenoestrogen effects are still controversial. This study aimed to investigate the effects of BPA characteristics and alternatives on osteoclast differentiation. Mouse osteoclast precursor RAW 264.7 cells were used for the in vitro osteoclast differentiation. BPA, BPF, and TMBPF showed no serious cytotoxicity in RAW 264.7 cells. In particular, we found TMBPF, a new BPA alternative chemical, to dramatically activate RANKL-induced osteoclast differentiation and induce mRNA levels of various osteoclast target genes, such as *NFATc1*, *TRAP*, and *CtsK*. Moreover, TMBPF activated osteoclast differentiation through activation of the MAPK signaling pathway, JNK, and p38. Our findings suggest that TMBPF, as a BPA alternative, also exerts xenoestrogenic effects, which have a negative impact on bone remodeling and skeletal homeostasis.

## Data Availability

The data presented in this study are available on request from the corresponding author.

## Sample Availability

Samples of the compounds are not available from authors.
